# A Primer on a Comprehensive Genetic Approach to Vascular Anomalies

**DOI:** 10.3389/fped.2020.579591

**Published:** 2020-10-19

**Authors:** Alexandra J. Borst, Taizo A. Nakano, Francine Blei, Denise M. Adams, Jessica Duis

**Affiliations:** ^1^Vascular Anomalies Program, Monroe Carrell Jr. Children's Hospital, Vanderbilt University Medical Center, Nashville, TN, United States; ^2^Vascular Anomalies Center, Children's Hospital Colorado, University of Colorado School of Medicine, Aurora, CO, United States; ^3^Vascular Anomalies Program, Lenox Hill Hospital, Northwell Health, New York, NY, United States; ^4^Vascular Anomalies Center, Children's Hospital of Philadelphia, University of Pennsylvania, Philadelphia, PA, United States

**Keywords:** vascular malformations, genetic, mutation, anomalies, somatic

## Abstract

The field of vascular anomalies has grown tremendously in the last few decades with the identification of key molecular pathways and genetic mutations that drive the formation and progression of vascular anomalies. Understanding these pathways is critical for the classification of vascular anomalies, patient care, and development of novel therapeutics. The goal of this review is to provide a basic understanding of the classification of vascular anomalies and knowledge of their underlying molecular pathways. Here we provide an organizational framework for phenotype/genotype correlation and subsequent development of a diagnostic and treatment roadmap. With the increasing importance of genetics in the diagnosis and treatment of vascular anomalies, we highlight the importance of clinical geneticists as part of a comprehensive multidisciplinary vascular anomalies team.

## Introduction

Vascular anomalies encompass both vascular malformations and vascular tumors (1). Over the past few decades, our knowledge of the genetic changes underlying these tumors and malformations has grown tremendously. This has led to updates in the classification of some lesions as well as new therapeutic options. The goal of this review is to provide teams taking care of children and adults with vascular anomalies an overview of the molecular pathways involved in disease pathogenesis and an organizational framework based on the current classification system that encompasses knowledge of their genetic and molecular etiologies. We also aim to provide a roadmap for evaluation of vascular anomalies based on both clinical and genetic features and to highlight the critical role of genetic diagnosis in the classification and treatment of vascular anomalies.

### Developmental Origin and Classification of Vascular Anomalies

Vascular malformations form due to errors in vascular morphogenesis, which occurs during early embryonic life, sometime between 4 and 10 weeks of gestation. Mesenchymal cells form primitive groups, called hemangioblasts, that differentiate into the four types of circulatory vessels (arterial, venous, capillary, lymphatic) through a series of remodeling, pruning, and continued growth. This is a process referred to as angiogenesis. The molecular mechanisms that govern angiogenesis are complex, with a series of genes controlling each critical step, from blood island formation, to endothelial cell differentiation, sprouting, migration, and remodeling (2, 3). Identifying and understanding these molecular mechanisms is key to the development of targeted therapeutics for children with vascular anomalies.

The care of patients with vascular anomalies has been plagued by misdiagnosis, which can delay appropriate treatment and misinform patients and their families. Comprehensive diagnostic evaluation requires careful attention to patient and family history, clinical and radiologic features of the lesion, and thoughtful classification of the lesion. Many centers utilize a multidisciplinary team, with both medical and surgical providers with experience and expertise in vascular anomalies, to provide comprehensive care for these patients. Increasingly, geneticists are becoming key members of such multidisciplinary teams. The important role of genetics in the care of patients with vascular anomalies is highlighted by updates to the classification system. In 1996, the International Society for the Study of Vascular Anomalies (ISSVA) developed a standardized classification guideline for children and adults with vascular Anomalies (4–6). In 2018, the ISSVA classification system was updated to capture our expanding knowledge base and report known genetic causes of vascular anomalies (6, 7).

### Inherited vs. Sporadic Vascular Anomalies

**Figure 1:**
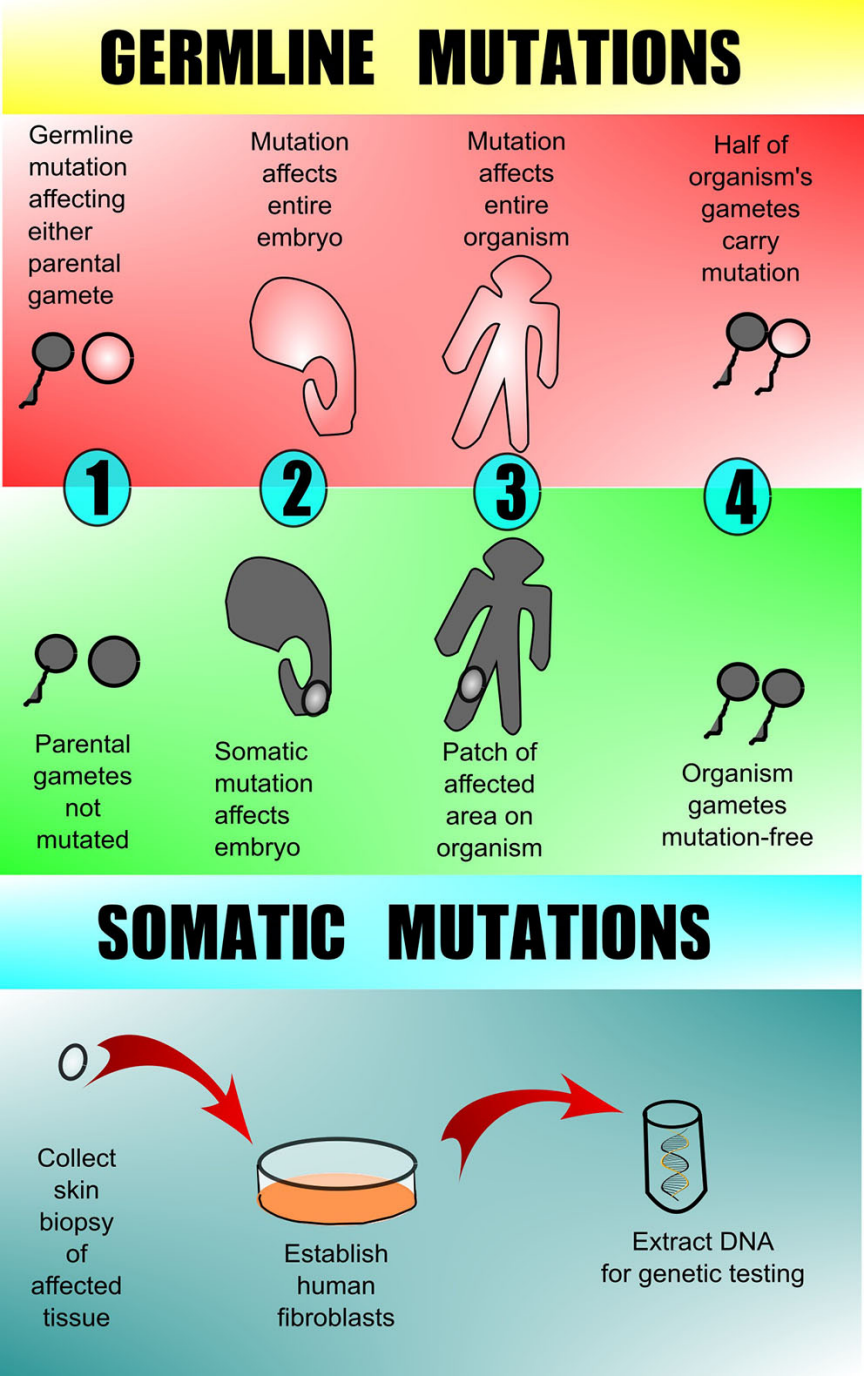
Germline vs. somatic mutations. Germline mutations are found in one or more of the parental gametes and thus subsequently affect all of the cells of the offspring. Somatic mutations arise in a non-gamete cell of the affected offspring and therefore are present in only 1 area or tissue type and are not in the offspring's gametes. Testing for germline mutations can be done on peripheral blood, but testing for somatic mutations requires biopsy of the affected tissue with subsequent DNA analysis. Peripheral blood and/or unaffected tissue may sometimes be sent along with affected tissue for comparison when searching for somatic mutation.

Understanding the types of genetic changes that drive the formation of vascular anomalies is key to appropriate testing and diagnosis. These pathogenic genetic variants can be categorized as either germline (familial or heritable mutations) or somatic (acquired mutation in somatic “body” tissues). The majority of vascular anomalies arise from somatic mutations, in which there is a post-zygotic mutation in a single cell that then perpetuates itself and is present in groups of cells throughout the body (called mosaicism). In this case, the mutation is not present in every cell. Genetic testing in patients with high suspicion for somatic driver mutations is presently best completed on directly affected tissue, which often requires surgical or skin biopsy from the vascular anomaly (Figure 1). Conversely, germline mutations are present in all of the individual's cells including the egg and sperm. Genetic testing in patients with high suspicion for germline driver mutations can be completed on any tissue, skin, blood, hair, or saliva.

Familial or inherited vascular malformations or syndromes are less common but provide unique insight regarding the molecular mechanisms that control vascular morphogenesis. The majority are inherited in an autosomal dominant fashion and involve mutations that lead to a loss-of-function of the affected gene. There is generally not 100% penetrance of familial vascular disorders and some of the phenotypes may not be readily apparent until later in life, suggesting that a second hit may be needed for tumor or malformation to present. In addition, a wide spectrum may be noted with respect to type, location, and presence of the malformation. When evaluating a patient with a vascular anomaly, family history including a 3-generation pedigree, may determine whether one might consider a germline mutation. Family and personal history of cutaneous findings, macrocephaly, history of malignancies, and developmental or learning disabilities; for example, implicates PTEN Hamartoma Tumor Syndrome (PHTS) and prompts germline testing to confirm. This has immediate implications for management with surveillance for thyroid cancer starting in childhood and additional malignancies in adulthood (8).

Historically, a low frequency of identified germline mutations led clinicians to seek tissue biopsy and many key somatic mutations were identified in this manner. Identification of tissue specific somatic mutations expands our understanding of the phenotypic spectrum of these disorders and our knowledge of their pathophysiology. As we continue to define the specific molecular changes and learn the breadth of clinical findings, we improve the inaccuracies in clinical classification and may be able to pursue genotype-phenotype correlation (9). Molecular diagnosis provides objective classification and serves to direct personalized therapies based on molecular phenotype (10). The identification of genes involved in these developmental defects of the vasculature is critical to our understanding of their etiology, as well as providing targeted therapeutic interventions (3, 11, 12).

### Role of Genetics

**Figure 2:**
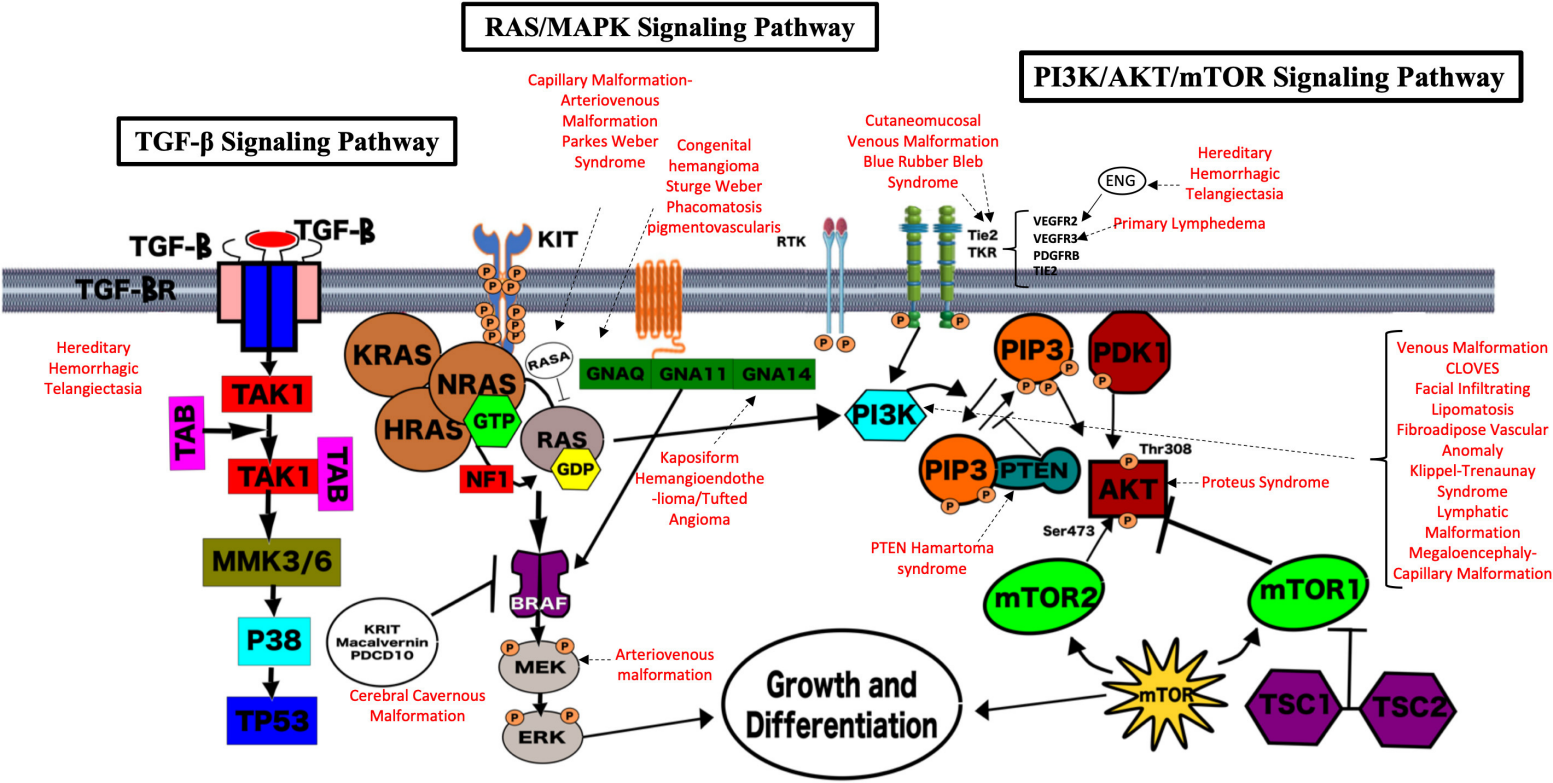
Genetic pathways implicated in vascular anomalies. The majority of identified mutations in vascular anomalies occur within two key intracellular signaling pathways—the RAS/MAPK and PI3K/AKT/mTOR pathways. The PI3K/AKT/mTOR pathway is crucial for many cellular processes, including cell cycle regulation, proliferation, and migration, and is often termed the “anti-apoptosis pathway.” Several activating mutations within this pathway are associated with vascular anomalies and complex vascular syndromes. The RAS/MAPK pathway is also crucial for cell cycle regulation, proliferation and migration, and is often referred to as the “proliferation pathway.” Several vascular anomalies are associated with mutations in this pathway and frequently termed “rasopathies.” There is also crosstalk between these 2 main pathways. The TGF-β Signaling Pathway is also key for regulation of numerous biological processes, has been implicated in the pathophysiology of hereditary hemorrhagic telangiectasia. Each pathway and their overlap are demonstrated pictorially here. The known associations with vascular malformations and syndromes are highlighted in red.

Genetic testing is now a key component of comprehensive care for patients with vascular anomalies. Accurate identification of a genetic variant can aid in definitive diagnosis, allow for necessary preventive screening, and directly impact therapeutic decision making. Elucidation of the genetic changes underlying vascular anomalies has also played an invaluable role in understanding normal vascular developmental programming and biology. The landscape has changed dramatically with the recognition of an expanding repertoire of germline and somatic mutations recognized to drive the development of vascular malformations and tumors. The majority of identified mutations in vascular anomalies occur within two key intracellular signaling pathways—the RAS/MAPK and PI3K/AKT/mTOR pathways (Figure 2). The PI3K/AKT/mTOR pathway is crucial for many cellular processes, including cell cycle regulation, proliferation, and migration, and is often termed the “anti-apoptosis pathway.” Several activating mutations within this pathway are associated with vascular anomalies and complex vascular syndromes. The RAS/MAPK pathway is also crucial for cell cycle regulation, proliferation, and migration, and is often referred to as the “proliferation pathway.” Several vascular anomalies are associated with mutations in this pathway and frequently termed “rasopathies.” There is also crosstalk between these 2 main pathways. Finally, TGF-β signaling, which is ubiquitous and key for regulation of numerous biological processes, has been implicated in the pathophysiology of hereditary hemorrhagic telangiectasia. As we learn more about the mutations involved in the development of various vascular malformations and tumors, it becomes clear that activating mutations in these key intracellular signaling pathways are frequently the cause of endothelial cell dysfunction and malformation development (13). The types of malformations or tumors that result are dependent on the specific mutation, tissue mosaicism, impact on protein function, and interaction with other genes. For example, mammalian target of rapamycin (mTOR) signaling is initiated in the phosphoinositide 3-kinase (PI3K)/AKT pathway involved in cell cycle regulation and often implicated in patients with vascular anomalies and overgrowth who have somatic mutations in *PIK3CA*. This finding has led to targeting of the overactive PI3K/AKT/mTOR pathway with sirolimus (an mTOR inhibitor), which has demonstrated clinical benefit in several vascular malformations and tumors (10, 11). As we continue to identify the specific driver mutations that cause mosaic or sporadic vascular anomalies, our understanding of the pathophysiology will continue to grow and offer insight into therapeutic strategies. It is very likely that additional gene mutations will be identified and become future targets for therapy. We offer here the current information regarding protein function for the known genetic mutations in vascular malformations and tumors. However, this information is growing rapidly and we are still at the very early stages of understanding this biology and identifying future targets for therapy that will continue to shape the management of vascular anomalies.

## Vascular Malformations

**Figure 3:**
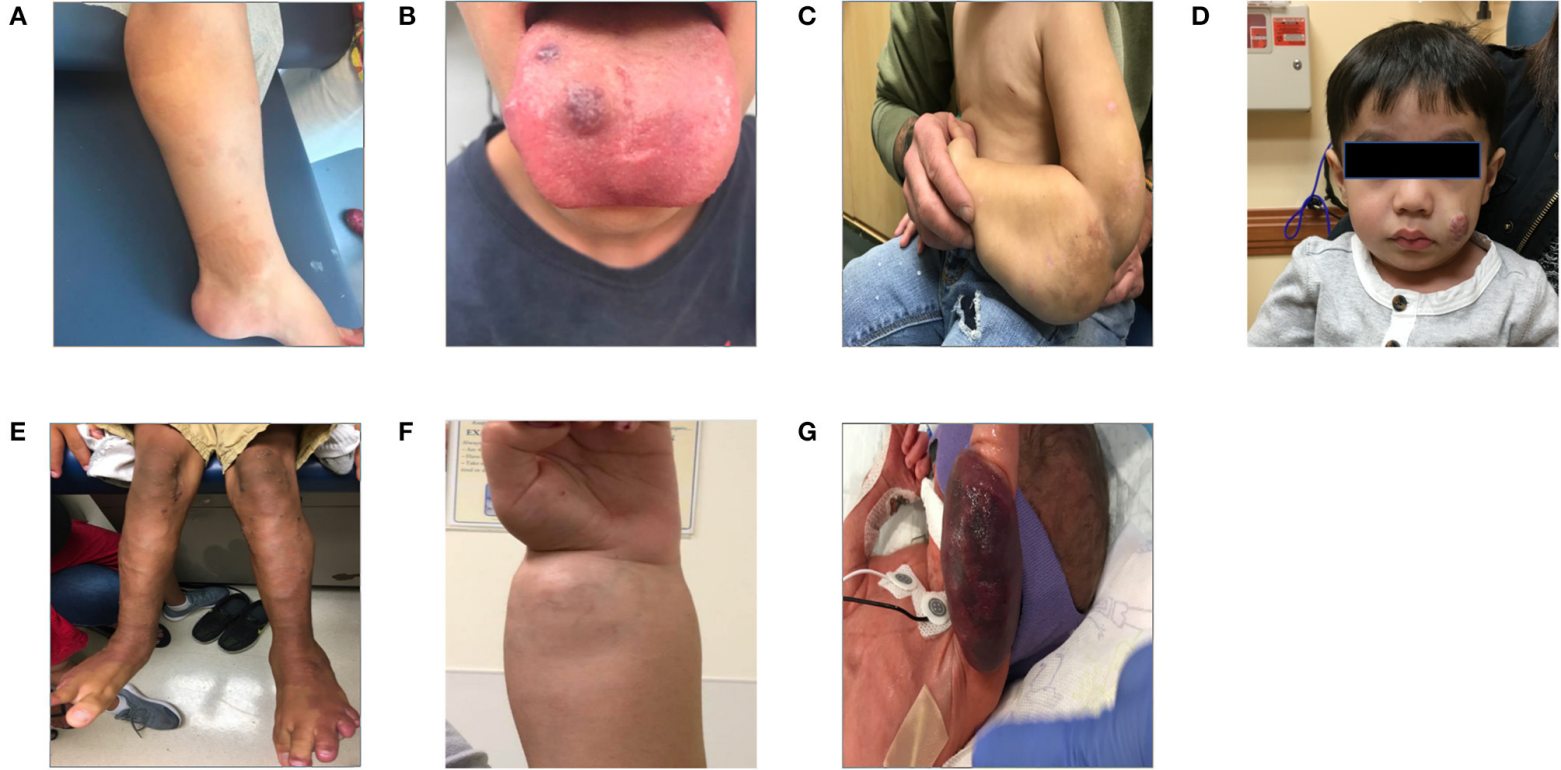
Patients with vascular anomalies. This panel of patient photographs shows a sampling of some of the physical exam findings in a few key disorders. **(A)** Shows a patient with the typical capillary malformations seen in patients with RASA1 mutation found on the lower extremity. **(B)** Shows a patient with Blue Rubber Bleb Nevus syndrome and the typical venous malformations seen here on the tongue. **(C)** Shows a patient with a large lymphatic malformation of the left upper extremity. The patient is several months into treatment with sirolimus. **(D)** Shows a young child with an infantile hemangioma of the cheek. **(E)** Shows the somatic overgrowth, capillary malformation, prominent superficial veins, and lymphangiomas in a patient with CLOVES syndrome. **(F)** Shows a patient with PTEN hamartoma syndrome and an intramuscular vascular malformation of the forearm/wrist. **(G)** Shows an infant with a Kaposiform hemangioendothelioma who presented with Kasabach-Merritt syndrome.

**Figure 4:**
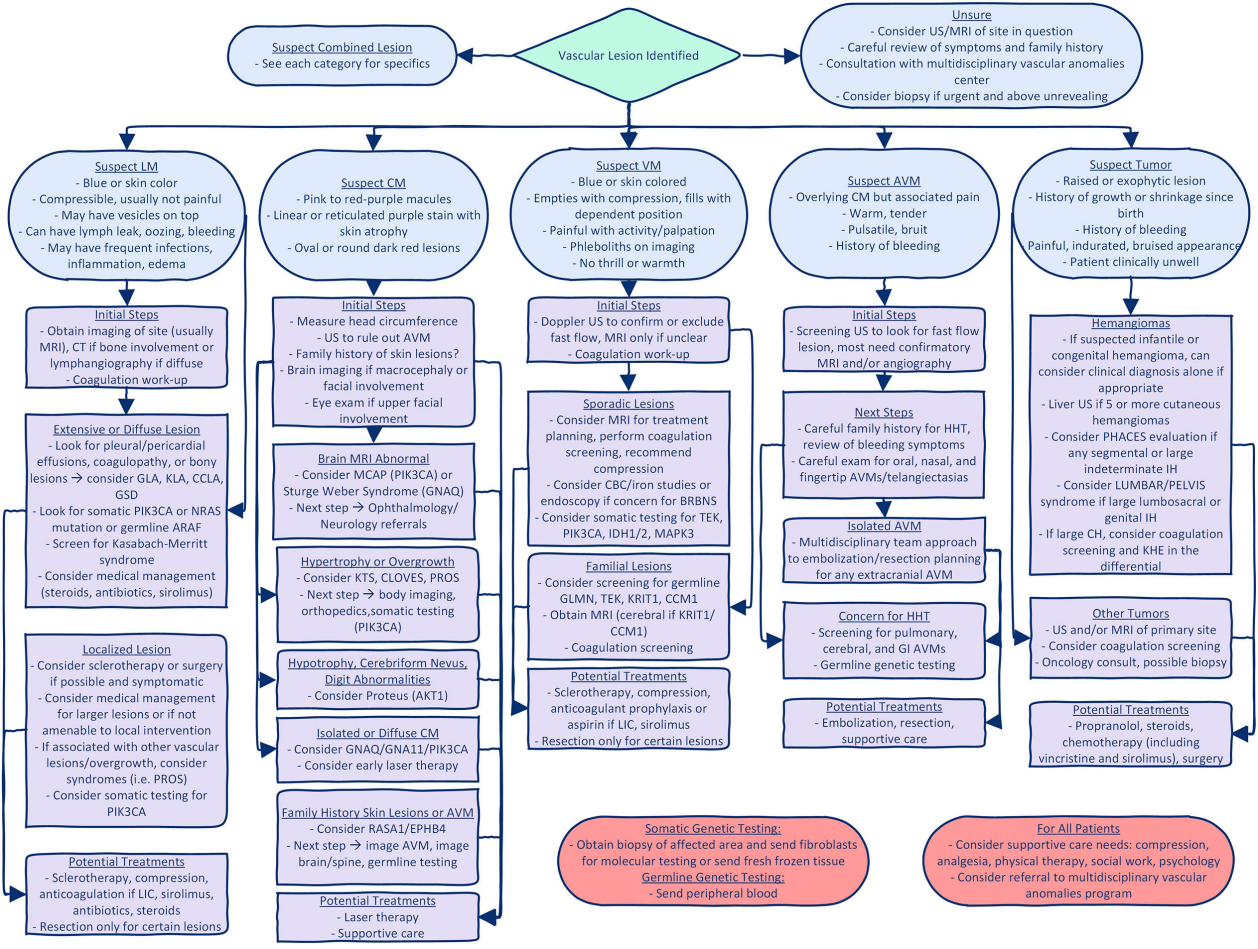
Diagnostic algorithm for vascular anomalies. This figure gives a suggested diagnostic algorithm for the initial evaluation and next steps when evaluating a patient with a vascular malformation or tumor. It is not all inclusive, but gives suggestions based on initial characteristics of the lesion and highlights key/critical steps that should not be missed. GLA, generalized lymphatic anomaly; KLA, Kaposiform lymphatic anomaly; CCLA, central conducting lymphatic anomaly; GSD, Gorham Stout Disease; LIC, localized intravascular coagulation; KTS, Klippel Trenaunay Syndrome; PROS, PIK3CA related overgrowth spectrum; BRBN, Blue Rubber Bleb Nevus; IH, infantile hemangioma; CH, congenital hemangioma.

Using the 2018 International Society for the Study of Vascular Anomalies classification of vascular malformation by simple subtype (6, 7), we provide an outline of the inherited/germline and somatic mutations identified in various malformations and recommended clinical evaluation for each malformation or syndrome. Examples of some of the key vascular malformations, syndromes, and tumors are found in Figure 3. We also provide a pathway for initial evaluation and recommended “next steps” for each type of vascular malformation or tumor (Figure 4). When guided by a careful history and physical exam, we believe this tool can be beneficial to clinicians as they navigate initial evaluation and management, recognizing that ultimately a multidisciplinary approach and opinion will be invaluable.

### Capillary Malformations

Capillary malformations (CMs), commonly known as port-wine stains, are common in the general population with an incidence of 0.3% (14). Pathologically they are defined by dilated venule-type channels in the superficial dermis with characteristically slow-flow on imaging. Capillary malformations are often associated with underlying soft tissue or bony hypertrophy, though whether this is causative or associated with the increased blood flow is unclear. They are typically present at the time of birth and may change in color over time. With age, vascular stasis in the abnormally formed capillaries results in vascular ectasia and soft tissue thickening. Although most isolated capillary malformations and associated syndromes are sporadic (Table 1), familial inheritance has been described (Table 2). Sturge-Weber syndrome (SWS) represents the most well-known cause of somatic capillary malformation. The capillary malformations seen in SWS and the more common isolated, uncomplicated capillary malformations are frequently found to have mutations in *GNAQ or GNA11*, which can lead to constitutive activation of the RAS/MAPK pathway (Figure 1) (24, 25). Inherited capillary malformation syndromes are most commonly due to mutations in the RAS/MAPK signaling pathway and are inherited in an autosomal dominant fashion with incomplete penetrance and variable expression.

**Table 1:** Somatic disorders involving capillary malformations.

**Associated malformation or syndrome**	**Clinical phenotype**	**Candidate gene**	**Protein function or suspected role (if known)**	**Clinical evaluation**	**References**
Sporadic CM (port wine stain)	• Primarily sporadic • Some AD inheritance • Pink stain at birth that darkens over time • Bony or soft tissue hypertrophy	Del-1 *GNAQ* *GNA11*	• Extracellular matrix. Organization of the formation and remodeling of blood vessels. Del-1 preserves mitotic state of proliferating cells. GNAQ/GNA11 mediate signals between G-protein-coupled receptors and downstream effectors. Increased RAS/MAPK signaling	• Physical exam • Developmental monitoring • Ophthalmologic exam • Brain MRI	(3, 9–12, 15)
Cutis marmorata telangiectasia (CMTC)	• Reticulated cutaneous stain (unilateral on the lower limb); apparent in low temperature or crying and improves over first year of life • Mistaken for livedo reticularis and telangiectasias • Hemihypertrophy and/or glaucoma	Unknown, with AD inheritance suggested in some cases. Possible *GNA11* implicated	• Abnormal pericyte recruitment causing skin capillaries to contract inappropriately	• Physical exam (musculoskeletal, vascular, cardiac, neurologic, skin, or eye abnormalities) • Rule out glaucoma, cutis aplasia, hypospadias • EKG (arrhythmia)	(3, 16, 17)
Sturge weber syndrome (SWS)	• Upper facial CM associated with ocular anomalies (glaucoma, choroidal vascular anomalies) • Leptomeningeal vascular anomalies • Seizures, developmental delay, contralateral hemiplegia • Soft tissue overgrowth (55–70%), and skeletal hypertrophy(maxilla)	*GNAQ*	• Mediates signals between G-protein-coupled receptors and downstream effectors. Increased RAS/MAPK signaling	• Whole body and brain MRI • Developmental screening • Ophthalmologic examination for glaucoma • Neurologic examination and EEG	(3, 9, 11, 15)
Phacomatosis pigmentovascularis	• Vascular and pigmented nevi present at birth and associated with scleral or intraocular melanocytosis • Glaucoma • Seizures • Cognitive delay	*GNA11* *GNAQ*	• Mediate signals between G-protein-coupled receptors and downstream effectors. Increased RAS/MAPK signaling	• Whole body/brain MRI • Ophthalmology • Assess development • EEG • Melanoma screen (eye structures)	(18, 19)

**Table 2:** Inherited/germline conditions involving capillary malformations (CM).

**Associated malformation or syndrome**	**Inheritance**	**Clinical phenotype**	**Candidate gene**	**Protein function or suspected role (if known)**	**Clinical evaluation**	**References**
Capillary malformation–arteriovenous malformation (CM–AVM)^*^	AD	• Circumscribed, small diffusely distributed CMs (6% single lesion); halo surrounding • AVM lesions (seen in 80%, intra- or extracranial) • May be associated with vein-of-Galen aneurysm	*RASA1* *EPHB4*	• Loss of function; encodes Ras GTPase activating protein p120RasGAP	• Brain/spine imaging • Genetic testing and counseling • Screening echo • X-rays for LLD • ENT-epistaxis. • US fast-flow lesion	(3, 9, 11, 15, 20, 21)
Generalized essential telangiectasia	AD	• Generalized telangiectasias that progress peripheral to central • Sporadic • Associated with mild pruritis, numbness, tingling, or burning • Conjunctival/mucosal telangiectasia • Diagnosis of exclusion (often white females)	None identified	• Unknown	• Rule out gastric bleeding	(3, 22)
Angioma serpiginosum	X-linked	• Classical pinpoint, dilated, think walled capillaries, located sub-epidermally along the lines of Blaschko	Unknown reports of Xp11.23 deletion containing *PORCN* (but contradicted by some)	• Unknown	• EGD to rule out esophageal papillomas • Skin and nail exam	(3, 23)

* *Note that CM-AVM is classified under both capillary and arteriovenous malformation. LLD, leg length discrepancy*.

### Venous Malformations

Venous malformations (VMs) are slow-flow lesions that can either be mass-like within tissues and unrelated to named veins, or they can represent anomalous development of anatomic veins. They are often considered the most common subtype of vascular malformation, with an incidence of between 1 in 5,000 to 1 in 10,000 births. They can range in size from very small to extensive and are purple to bluish on color due to stagnant blood flow within the lesion, that contributes to pain, swelling, and thrombotic complications (4, 5, 26–28). The majority of lesions are noted at birth, but deeper lesions are sometimes asymptomatic until the time of puberty when they tend to increase in size and become symptomatic. More than 90% of venous malformations are sporadic and isolated in nature (Table 3), but may rarely present with a familial pattern (Table 4) (15). TIE2/TEK gene mutations are thought to be causative in most isolated venous malformations. However, more recently, somatic activating mutations in PIK3CA have also been identified in venous malformations (41). TIE2/TEK is an endothelial cell specific tyrosine kinase receptor that functions via the PI3K/AKT/mTOR signaling pathway (Figure 1) and plays a significant role in regulating angiogenesis, proliferation, cell migration, and vessels stability (3, 15). Blue rubber bleb nevus syndrome (BRBNS) is a rare, often severe disorder known to be caused by TIE2/TEK somatic mutations (30). Careful documentation of birthmarks on family pedigree can help identity hereditary cases. Glomuvenous malformation and mucocutaneous venous malformation (VMCM) have demonstrated autosomal dominant inheritance but are thought to require a second hit for lesion formation (26).

**Table 3:** Somatic disorders involving venous malformations.

**Associated malformation or syndrome**	**Clinical phenotype**	**Candidate gene**	**Protein function or suspected role (if known)**	**Clinical evaluation**	**References**
Sporadic VMs	• Solitary, localized, and non-familial • Soft, mass or sponge-like lesion with blue or purple coloration	*TIE2/TEK* *PIK3CA*	• Endothelial cell-specific tyrosine kinase receptor. Important for angiogenesis (angiopoietin receptor). Gain of function mutations, probably affects endothelial cell behavior, may involve paracrine signaling between endothelial and smooth muscle cells, down-regulation of PDGF-beta production, only mucocutaneous veins are affected • Catalytic alpha subunit of PI3K. Somatic activating mutation that increases PI3K/AKT/mTOR signaling	• US or MRI of lesion depending on location and symptoms • Screening for localized intravascular coagulopathy (PT, PTT, fibrinogen, d-dimer) • Consider brain MRI	(3, 10–12, 15)
Multifocal VM	• Rare, multifocal variant • Tend to be mosaic for first mutation with 2nd hit	*TIE2/TEK*	• Endothelial cell-specific tyrosine kinase receptor. Important for angiogenesis (angiopoietin receptor)	• Consider whole body MRI	(11, 12)
Verrucous VM	• Raised reddish-purple hyperkeratotic lesion (extremities) • Enlarge over time, more hyperkeratotic with increased bleeding	*MAP3K3*	• Somatic activating mutation that may increase RAS/MAPK signaling	• Skin exam • Consider evaluation for underlying bleeding diathesis	(3, 10, 12, 29)
Fibroadipose vascular anomaly (FAVA)	• Within a muscle; increased fibroadipose tissue and smaller, non-spongiform vessels • Often single limb, appears slightly enlarged	*PIK3CA*	• Catalytic alpha subunit of PI3K. Somatic activating mutation that increases PI3K/AKT/mTOR signaling	MRI or US of the lesion	(3, 10, 12)
Blue rubber bleb nevus syndrome	• Sporadic, some AD inheritance • Multiple cutaneous and internal VM • Intestinal and hepatic • Tens to hundreds of blue or purple, compressible, and hyperkeratotic	*TEK/TIE2*	• Endothelial cell-specific tyrosine kinase receptor. Important for angiogenesis (angiopoietin receptor)	• MRI or ultrasound, consider full body MRI consider biopsy • Screening for localized intravascular coagulopathy (D-dimer, fibrinogen, PT, PTT) • Urinalysis for hematuria • Screening for iron deficiency anemia • Endoscopy/colonoscopy	(3, 10–12, 15, 30, 31)
Cerebral cavernous malformation (CCM)	• Immature vessels reflecting abnormal angiogenesis presents with seizures, headaches, hemorrhage, and neurologic defects	*KRIT1 (CCM1)* *CCM2* *CCM3* *Sporadic or AD*	• Adaptor protein Integrin β1 pathway involved in arterial specification, cell adhesion, endothelial cell junctions, and migration • Adaptor protein, scaffold protein, overlaps with KRIT1 • Apoptosis, VEGF signaling. Overlaps with KRIT1 • The CCM proteins interact together and dysfunction of the CCM signaling complex leads to altered vascular integrity and endothelial cell organization. They suppress RhoA GTPase signaling	• Brain MRI with gradient echo or susceptibility weighted imaging	(3, 9–12, 15)

**Table 4:** Inherited/germline conditions involving venous malformations.

**Associated malformation or syndrome**	**Clinical phenotype**	**Candidate gene/inheritance**	**Protein function or suspected role (if known)**	**Clinical evaluation**	**References**
PTEN-associated venous anomalies	• Hamartoma of soft tissue, intramuscular vascular lesions, with fast-flow lesions in 86% • Increased risk of cancer	*PTEN* AD	• Tumor suppressor	• Head circumference • Skin exam • Brain MRI with contrast • Cancer screening (thyroid, breast, uterine, colon)	(3, 32–34)
Mucocutaneous venous malformations (VMCM)	• Small, multifocal bluish muco-cutaneous lesions	*TIE2/TEK* AD	• Endothelial cell-specific tyrosine kinase receptor. Important for angiogenesis (angiopoietin receptor)	• Physical exam, consider imaging • Consider cardiac work up	(3, 15, 35)
Cerebral cavernous malformation, familial	• Cerebral lesions dilated channels with endothelial cell layers that have defective tight junctions in the brain, retina and spinal cord • Majority present in between the second and fifth decades with seizures, focal neurologic findings, headaches, and cerebral hemorrhage • Cutaneous vascular lesions in 9% and retinal vascular lesions in 5%	*KRIT1* *CCM2* *PDCD10* AD	• Loss-of-function mutations affecting subendothelial matrix, vascular structure, and adhesion	• Brain MRI with gradient echo or susceptibility weighted imaging	(3, 36)
Glomuvenous malformations or Glomangiomas	• Superficial, multiple raised or plaque-like lesions • Cobblestone appearance, painful on palpatio	*GLMN* AD, 100% penetrance with variable expressivity	• Phosphorylated protein that is a member of the Skp1-Cullin-F-box-like complex • Essential for normal development of the vasculature • Loss of function mutation, likely requires a somatic second hit	• Detailed skin exam • Genetic testing and counseling • Biopsy vs. removal if concern about diagnosis • No routine imaging unless clinical concern	(3, 15, 26, 37)
Hyperkeratotic cutaneous capillary-venous malformation (HCCVM)	• Crimson-colored irregularly shaped lesions that extend into the dermis and hypodermis • Composed of dilated capillaries and blood-filled venous-like channels • Associated with cerebral capillary malformations, which may present with headaches, seizures, and intracranial hemorrhag	*KRIT1* (also known as CCM1) *CCM2* *CCM3* Possible AD	• RAS antagonist, may be involved in cellular adhesion and vascular integrity. The CCM proteins interact together and dysfunction of the CCM signaling complex leads to altered vascular integrity and endothelial cell organization	• Detailed skin exam • Brain imaging for cerebral capillary malformations	(38, 39)
Varicose veins	• Twice as common in females as males	*FOXC2* AD, with reduced penetrance	• Unkown	• Physical exam with referral to vascular surgeon if symptomatic concerns	(3, 40)

### Lymphatic Malformations

Lymphatic malformations (LM) encompass a range of developmental or functional defects affecting lymphatic vessels. These can be focal or diffuse disorders with marked heterogeneity (Table 5) (46). Lymphatic anomalies can be associated with significant comorbidities such as pain, infection, visceral, and bone involvement and disfigurement. Most lymphatic malformations are due to sporadic mutations in genes that regulate lymphangiogenesis. However, certain genetic syndromes are associated with developmental abnormalities of the lymphatics, including Down syndrome, Turner syndrome, Noonan syndrome and Cardiofaciocutaneous syndrome (47, 48). These are important to be aware of in the general evaluation of children with these disorders. Lymphedema is clinically distinct from lymphatic malformations in that it is due to functional defects in initial or collecting lymphatics. Some forms of lymphedema and related syndromes appear to have familial inheritance (Table 6) (3). Sporadic lymphatic malformations and related syndromes are believed to involve mutations in VEGFC/VEGFR3 and PI3K/AKT/mTOR pathways, but a clear pathogenetic mechanisms has not been defined (3, 15).

**Table 5:** Somatic disorders involving lymphatic malformations.

**Associated malformation or syndrome**	**Clinical phenotype**	**Candidate gene**	**Protein function or suspected role (if known)**	**Clinical work-up**	**References**
Sporadic LMs	• Localized or extensive malformed lymphatic vessels in the skin or deep soft tissues • Predilection for head/neck areas	*PIK3CA*	• Catalytic alpha subunit of PI3K. Somatic activating mutation that increases PI3K/AKT/mTOR signaling	• Imaging of involved site with MRI • Evaluation for other sites of disease if clinically indicated	(3, 10–12)
Generalized lymphatic anomaly (GLA)	• Previously known as lymphangiomatosis • Diffuse or multifocal LMs throughout the skin, soft tissues, abdominal, and thoracic viscera • Pericardial and pleural effusions may be noted • May have significant bone involvement	*PIK3CA*	• Catalytic alpha subunit of PI3K. Somatic activating mutation that increases PI3K/AKT/mTOR signaling	• Full body imaging with CT and/or MRI • Screening for coagulopathy (PT, PTT, fibrinogen, d-dimer) • Biopsy not necessary • Echo	(10, 11, 42)
Gorham stout syndrome, aka “disappearing bone disease”	• Progressive osteolysis with replacement of bone by soft tissue and vascular channels, primarily lymphatic in origin • Defining characteristic is disappearance of bone rather than a mass lesion in bone	Unknown	• Likely involvement of PI3K/AKT/mTOR signaling pathway	• Full body imaging with CT and/or MRI • Screening for coagulopathy (PT, PTT, fibrinogen, d-dimer) • Biopsy not necessary • Echo	(3, 42, 43)
Kaposiform lymphangiomatosis (KLA)	• Systemic and frequently aggressive lymphatic anomaly • More extensive thoracic involvement than GLA • Frequently associated with coagulopathy, effusions, and intralesional hemorrhage • Systemic involvement is common	*NRAS*, possible	• Intracellular RAS signaling. A proto-oncogene that encodes a small GTPase that regulates cell proliferation in the RAS/MAPK and PI3K/AKT/mTOR signaling pathways	• Full body imaging with CT and/or MRI • Consider echocardiography • Screening for coagulopathy (PT, PTT, fibrinogen, d-dimer) • Consider angiopoietin and VEGFR3 screening • Biopsy not necessary	(3, 10, 11, 44, 45)
Congenital pulmonary LM	• LMs in the lungs, heart, pancreas, kidneys, and mesentery • Respiratory distress at birth due to pulmonary hypoplasia secondary to chylous pleural effusions • May overlap with Noonan, Turner, and Down syndromes	*CCBE1*	• Extracellular guidance molecule for migrating lymphatic endothelial cells, enhances lymphangiogenic activity of VEGF-C	• Full body imaging with CT and/or MRI • Consider echocardiography • Developmental and neurologic screening	(3, 15)

**Table 6:** Inherited/germline conditions involving lymphatic malformations.

**Associated malformation or syndrome**	**Inheritance**	**Clinical phenotype**	**Candidate gene**	**Protein function or suspected role (if known)**	**Clinical evaluation**	**References**
Primary lymphedema (Nonne-Milroy disease)	AD	• Lower-limb lymphedema, present as pedal edema at birth or soon after • Usually bilateral • Hydrocele (males) • Prominent veins • Upslanting toenails • Papillomatosis • Urethral anomalies in males	*FLT4* *VEGFR3*	• Endothelial cell tyrosine kinase receptor, important for lymphangiogenesis (VEGF-C receptor) • Transcription factor, regulates PDGFβ	• MR lymphangiography or NM lymphoscintigraphy (lack of uptake radioactive colloid in ilioinguinal lymph nodes) • PT/OT evaluation • Compression fitting • Good skin hygiene • Genetic testing and counseling	(3, 10, 11, 15)
Late-onset lymphedema (Meige Disease)	Probable AD	• Most common subtype of primary lymphedema (~80% all cases) • Present at birth but onset of symptoms at puberty • Edema begins in feet and ankles and progresses to legs and knees	*FOXC2* *GJC2*	• Transcription factor, regulates PDGFβ• Gap junction protein	• Evaluation as per primary lymphedema plus • Evaluation to rule out secondary lymphedema (malignancy, thrombosis, etc.)	(3)
Microcephaly-lymphedema-chorioretinal dysplasia	AD, or sporadic	• Patients have persistence of fetal lymphedema • Hypotonia, • Microcephaly • Intellectual disability • Chorioretinopathy	*KIF11*	• Spindle motor protein, may affect microtubule function	• Evaluation as per primary lymphedema plus • Ophthalmology exam • Echocardiogram • Consider brain MRI (lissencephaly reported)	(3, 15)
Hypotrichosis-lymphedema-telangiectasia syndrome	AR	• Absent eyebrows and eyelashes • Eyelid edema from birth • Lymphedema in the first of second decade of life • Telangiectasias (palms, soles, scalp, scrotum, legs)	*SOX18*	• Spindle motor protein, may affect microtubule function	• Evaluation as per primary lymphedema plus • Serial echocardiogram (dilation of the ascending aorta reported) • Skin and nail exam	(3, 15)
Lymphedema with distichiasis	AD	• Late onset lymphedema with distichiasis (fine hairs from Meibomian glands on inner eyelid) • Variable cardiac/renal/palate defects, varicose veins • Neck webbing	*FOXC2*	• Transcription factor, regulates PDGFβ	• Evaluation as per primary lymphedema plus • Ophthalmology exam (corneal irritation, recurrent conjunctivitis, and photophobia, ptosis) • Echocardiogram to rule out congenital heart disease • Spine MRI to rule out extradural arachnoid cysts	(3, 49)
Lymphedema with choanal atresia	AR	• Exceedingly rare, 1 family with lymphedema and choanal atresia	*PTPN14*	• Loss of function mutation in protein tyrosine phosphatase	• Evaluation as per primary lymphedema	(3)
Cholestasis-lymphedema (Aagenaes Syndrome)	AR	• Jaundice at birth and recurrent through life • Can progress to cirrhosis • Late-onset lower extremity lymphedema • Identified in several Norwegian families	Unknown gene on 15q	• Unknown	• Evaluation as per primary lymphedema plus • Hepatic/GI evaluation including CMP, serum bile acids (elevated), and lipid panel (hyperlipidemia)	(3)
Lymphedema-intestinal LM-mental retardation (Hennekam Syndrome)	AR	• Severe progressive lymphedema including genitalia and face • Mild to severe intellectual disability • Hypoproteinemia • Hearing loss • Renal anomalies • Scoliosis and club feet • Microcephaly ad craniosynostosis	*CCBE1* *FAT4* *ADAMTS3*	• Function in migration of lymphatic endothelial cells	• Evaluation as per primary lymphedema plus • Neurologic evaluation • Hearing screen • Abdominal US • Nutritional assessment • Thyroid studies • Screening for anemia • Spine X-rays	(3, 50–52)
Lymphedema-myelodysplasia (Emberger syndrome)	AD	• Lymphedema of limbs and genitalia in early childhood • Myelodysplasia with development of AML • Craniofacial anomalies • Congenital deafness	*GATA2*	• Transcription factor involved in gene regulation during vascular development and hematopoietic differentiation	• Evaluation per primary lymphedema plus • Hematologic evaluation including bone marrow biopsy • Neurologic and hearing assessments	(3, 53)

### Arteriovenous Malformations

Arteriovenous malformations (AVMs) occur due to a direct connection between an artery and vein, bypassing the normal capillary bed, leading to arterialization of the veins and a high-flow lesion with propensity to grow and bleed (54). Sporadic AVMs (Table 7) are commonly associated with mutations in the RAS/MAPK pathway (Figure 1). *RASA1* mutations are associated with CM-AVM and may be causative in some sporadic AVMs (3, 15). Other known molecular changes found in sporadic AVMs are found in MAP2K/MEK and several groups are starting to use MEK inhibitors for off-label management (29, 57). The most common inherited AVM syndrome (Table 8) is hereditary hemorrhagic telangiectasia (HHT), also known as Osler-Weber-Rendu syndrome. HHT is a disorder of vascular dysplasia characterized by multiple AVMs. Small telangiectasias may manifest as superficial skin and mucosal membrane lesions with potential to rupture presenting as epistaxis or gastrointestinal bleeding. Larger AVMs can be found within the lungs, central nervous system, and liver. HHT is inherited in an autosomal dominant manner with clinical heterogeneity and an age-dependent natural history. Pathologic mutations to genes involved in TGF-beta signaling pathway (Figure 1), important for angiogenesis, have been described including *ENG, ALK1/ACVRL1, GDF2, SMAD4/MADH4*. An appropriate pediatric HHT screening evaluation must include family history, physical examination, and a low threshold to proceed with non-invasive screening and imaging (58, 59). Heterozygous *ENG* and *ALK* mutations, such as found in HHT, have also been found in sporadic AVMs.

**Table 7:** Somatic disorders involving arteriovenous malformations.

**Associated malformation or syndrome**	**Clinical phenotype**	**Candidate gene**	**Protein function or suspected role (if known)**	**Clinical work-up**	**References**
Sporadic AVM	• Abnormal connections between arteries and veins without a normal capillary bed	*MAP2K1/MEK1* *KRAS*	• Stimulates enzymatic activity of MAP kinases	• Imaging with MRI and/or angiography	(3, 10, 11, 54–56)
	• Results in arterialization of venous system, pain, tissue destruction, and bleeding complications	*NRAS* *BRAF*	• Intracellular RAS/MAPK signaling	• Thorough exam/history to exclude HHT or CM-AVM	

**Table 8:** Inherited/germline conditions involving arteriovenous malformations.

**Associated malformation or syndrome**	**Inheritance**	**Clinical phenotype**	**Candidate gene**	**Protein function or suspected role (if known)**	**Clinical work-up**	**References**
Hereditary Hemorrhagic Telangiectasia (HHT) aka Osler-Weber-Rendu Syndrome	AD	• Telangiectasias (lip, tongue, buccal mucosa, face, chest, and fingers) • AVMs throughout multiple sites including cerebral, pulmonary, gastrointestinal, and hepatic • Most common clinic manifestation epistaxis (starting age 12 usually) • Iron deficiency anemia • Heart failure • Stroke • GI bleed 25%, commonly after 50	ENG, ALK1/ACVRL1, GDF2 SMAD4/MADH4	• TGF-β signaling pathway	• CBC/iron studies • Sitting and supine pulse oximetry every 1–2 years during childhood • Contrast echo every 5 years by age 10 • Periodic endoscopy/colonoscopy to rule out GI polyps and malignant change of juvenile polyps • ENT evaluation • Brain MRI/MRA in infancy and after puberty • Liver US • Genetic testing and counseling • Antibiotic prophylaxis for dental and invasive procedure • Filter on IV lines to prevent bubbles if pulmonary AVM is present	(3, 58, 59)
CM-AVM1^*^	AD or sporadic	• Small, multifocal CMs often accompanied by a pale halo • 30% of cases associated with additional deeper, fast-flow AVMs	*RASA1* *EPHB4*	• Loss of function; encodes Ras GTPase activating protein p120RasGAP	• Brain/Spine imaging • Genetic testing and counseling • Screening echocardiography • X-rays for leg length discrepancy • ENT evaluation if epistaxis • US shows fast-flow lesion	(3, 9, 11, 15, 20, 21)
CM-AVM2^*^	Unknown, likely AD	• Small telangiectasias around the lips and on upper thorax • Less frequently cerebral AVMs	*EPHB4*	• Loss of function mutation. Transmembrane receptor preferentially expressed in endothelial cells, acts via RAS/MAPK pathway	• Brain/Spine imaging • Genetic testing and counseling • Screening echocardiography • X-rays for leg length discrepancy • ENT evaluation if epistaxis	(12, 20, 21)

* *Note that CM-AVM is classified under both capillary and arteriovenous malformation*.

### Complex Syndromes Associated With Vascular Anomalies

There are several complex syndromes that include overgrowth of soft tissues in combination with vascular malformations. The majority of these syndromes are caused by somatic mutations, primarily isolated to the PI3K/AKT/mTOR signaling pathway (Figure 1) (11, 15). These syndromes and the candidate genes involved in their development are summarized in Table 9. Recognition of these syndromes is important due to the association of other complications, such as malignancy in CLOVES and Maffuci syndromes, and thrombosis in Klippel-Trenaunay and Proteus syndromes.

**Table 9:** Syndromes associated with vascular anomalies.

**Syndrome**	**Clinical phenotype**	**Candidate gene**	**Protein function or suspected role (if known)**	**Clinical work-up**	**References**
CLOVES syndrome	• Characterized by congenital lipomatous overgrowth • Vascular malformations • Epidermal nevi • Skeletal anomalies	*PIK3CA*, due to mosaic or missense mutations	• Catalytic alpha subunit of PI3K. Somatic activating mutation that increases PI3K/AKT/mTOR signaling	• Brain MRI • X-rays for leg length discrepancy • Scoliosis screen • Echocardiogram • EEG • Renal US to rule out anomalies and q3-6 months to screen for Wilms tumor • Screening for paraspinal high-flow lesions with spinal cord ischemia • Thrombophilia evaluation • Developmental • Feeding assessment	(3, 15)
Klippel-Trenaunay syndrome	• Characterized by slow-flow capillary-lymphatic-venous malformations and soft tissue overgrowth of an extremity and/or trunk • Often involves the pelvis as well. Can be unilateral or bilateral	*PIK3CA*, some cases *AGGF1*	• Catalytic alpha subunit of PI3K. Somatic activating mutation that increases PI3K/AKT/mTOR signaling • Angiogenic factor with G patch and FHA domains, increases angiogenesis *in vitro*	• MRI imaging of affected area • Thrombophilia evaluation and labs for localized intravascular coagulation	(3, 15)
Megalencephaly-capillary malformation syndrome (MCAP)	• Congenital megalencephaly or hemimegalencephaly • Reticulate capillary stains • CM of the lip/philtrum • Asymmetry, focal, or generalized overgrowth • Hypotonia • Seizures • Mild to severe intellectual disability • Syndactyly	*PIK3CA*, from brain tissue	• Catalytic alpha subunit of PI3K. Somatic activating mutation that increases PI3K/AKT/mTOR signaling	• Brain MRI q 6 months for the first 2 years then yearly till 8 years to rule out neurological complications • X-rays for leg length discrepancy • Scoliosis screen • EEG • Echocardiogram • Renal US to rule out anomalies and q 3 months to screen for Wilms tumor • Developmental and feeding assessment • Sleep evaluation • Thrombophilia evaluation	(3, 10, 15)
Maffucci syndrome	• Multiple spindle cell hemangiomas associated with multiple enchondromas • Vascular lesions often do not appear until puberty • Patients have increased risk for malignancy	*IDH1* and *IDH2*	• Mutant enzymes catalyze the reduction of alfa-ketoglutarate to D-2-hydroxyglutarate, cause downstream genomic hypermethylation	• Screening of lesions due to malignancy potential • Risk of many types of malignancy reported (chondrosarcomas, gliomas, ovarian tumors, and other sarcomas) • Limb length x-rays and imaging of the extremities for other malformations	(3, 10, 60)
Proteus syndrome	• Bony and soft tissue overgrowth that develops and progresses rapidly in the toddler period and tends to plateau after adolescence • Increased risk of malignancy • Pulmonary complications • Increased risk thrombus	*AKT1*	• Intracellular PI3K/AKT/mTOR signaling/apoptosis	• Scoliosis screen • Skin exam • Thrombophilia evaluation and monitoring for DVT and PE • Developmental assessment • Monitoring for bullous pulmonary disease	(3, 10, 11)
Parkes weber syndrome	• Similar to CM-AVM but with overgrowth of affected limb	*RASA1*, loss of function mutation	• Intracellular signaling, RasGTPase	• Brain/spine MRI • US shows fast-flow lesion	(3, 9, 11, 15)
Familial intraosseous vascular malformation	• Extensive vascular lesions in the intraosseus spaces of the craniofacial bones associated with other midline defects • AR inheritance • Intraosseous hemangioma often in the vertebral column or the skull • Most commonly affected bones are the mandible and the maxilla • Life threatening progressive expansion of the jaw • Craniofacial and other intramembranous bones caused by malformed blood vessels	*ELMO2*	• Translation of extracellular signals to cellular movements	• MRI head and neck • Midline screen to look for diastasis recti, supraumbilical raphe and hiatal hernia	(3, 61)

### Vascular Tumors

Vascular tumors include both benign, locally aggressive, and malignant tumors comprised of one or more vascular components (7, 62). The molecular etiology of many of these tumors is unknown, but some candidate genes have recently been identified (Table 10). Infantile hemangiomas are the most common vascular tumor, and are generally benign, but growth can be disruptive of function or cosmesis, and occasionally life-threatening. The role of the pediatrician is key to determining which lesions require referral for additional management. Vascular tumors other than hemangiomas often require multi-modal therapy, including chemotherapy, and consultation with a pediatric hematologist-oncologist is indicated.

**Table 10:** Vascular tumors.

**Tumor**	**Clinical phenotype**	**Candidate gene**	**Protein function or suspected role (if known)**	**Clinical work-up**	**References**
Infantile hemangioma	• Most common tumor of infancy • Comprised of capillaries and proliferating endothelial cells • Grow rapidly during first few months of life then regress in early childhood	Possible variants in *VEGFR2* and *TEM8*	• Altered VEGF-A and VEGFR2 signaling Constitutive activation of VEGF-dependent VEGFR2 signaling • Sequestration of integrin 1B, inhibition of NFAT transcription and reduction of VEGFR1	• Consider liver US if > 5 cutaneous hemangiomas • Consider evaluation for PHACES if facial segmental hemangioma (brain MRI/MRA, Echocardiogram, eye exam)	(3, 10, 12, 62)
Congenital hemangioma	• Fully formed at birth • Can be rapidly involuting (RICH), partially involuting (PICH), or non-involuting (NICH)	*GNAQ* *GNA11*	• Organization of the formation and remodeling of blood vessels. GNAQ mediates signals between G-protein-coupled receptors and downstream effectors Increased RAS/MAPK signaling	• None unless clinically indicated • Large lesions can have transient Kasabach-Merritt phenomenon (screen with CBC, fibrinogen, PT, PTT)	(3, 10, 11)
Pyogenic granuloma	• Post-natal lesion with mean onset around age 6 years • Benign, pedunculated, fragile, and frequently bleed	*KRAS, NRAS, HRAS* *GNAQ, BRAF*	• Upregulated RAS/MAPK/ERK signaling	• None unless clinically indicated	(3, 10, 11)
Kaposiform hemangioendothelioma and Tufted angioma	• Vascular neoplasm generally present at birth and enlarges during infancy • Locally aggressive. Associated with Kasabach-Merritt phenomenon	*GNA14*	• G-protein related signal transduction	• Evaluation for Kasabach Merritt phenomenon (CBC, fibrinogen, PT, PTT) • Imaging of site (MRI) • Oncology consult	(3, 10, 11)
Angiosarcoma	• High-grade malignant neoplasm of endothelial cell origins • Can arise in skin, deep soft tissues, or visceral organs • Can be radiation or lymphedema-associated	*PTPRB*, PLCG, KDR/VEGFR2 mutations, FLT4/VEGFR3 amplifications *MYC* amplification (in XRT induced)	• Vascular endothelial growth factor receptors • Proto-oncogene, increased expression of genes involved in cell proliferation	• Imaging of site and for metastatic disease, including brain • PET/CT • Oncology consult	(3, 10, 63, 64)
Epithelioid hemangioendothelioma	• Malignant endothelial tumor with variable clinical behavior • Multifocal lesions can be stable, grow slowly, or rapidly progress and metastasize	*WWTR1-CAMTA1* translocation *YAP1-TFE3* translocation	• Transcription factor signaling in the hippo pathway	• Imaging of site and for metastatic disease, including brain • Consider PET/CT • Oncology consult	(3, 10, 63, 65)
Familial infantile myofibromatosis	• Fibrous tumor of early childhood • Solitary lesions can regress • Multifocal or generalized lesions can be life threatening	*PDGFRβ*	• Receptor tyrosine kinase and mitogen for mesenchyme-derived cells, signaling in embryonic development, including recruitment of vascular smooth muscle cells	• Imaging of site and for metastatic disease • Oncology consult	(3, 10, 66, 67)

## Discussion

It is critical for teams treating patients with vascular anomalies to have a basic understanding of the genetic changes underlying vascular anomalies. Genetic results have implications for patient management, screening, and ultimately treatment.

As we begin to understand more about the genetic underpinnings of vascular anomalies, we identify potential therapeutic targets that will ultimately change treatment paradigms. Prior to the discovery of the underlying molecular pathways involved in the pathogenesis of vascular malformations and tumors, medical management was based primarily on symptom management with antiplatelet and anticoagulant agents to control localized intravascular coagulation and platelet activation, and pain medications to alleviate symptoms (1, 5, 8, 9). Over the years, multiple medications, primarily based on adaptation of oncologic agents, have been utilized in patients with complex and life-threatening vascular anomalies. This includes drugs such as bevacizumab, interferon-alpha, and cyclophosphamide, that have come with their own significant risks and side effects and generally can be considered sledgehammer rather than target approaches (68–70). Even propranolol's efficacy in infantile hemangiomas was identified serendipitously in children who received the drug for the cardiac complications they developed due to their massive infantile hemangiomas (71). Despite its successful use, the mechanism and relationship of propranolol to vascular growth pathways remains to be elucidated (72–74).

In the early 2000s, several groups began to report on the importance of PI3K/AKT/MAPK, and TGF-β signaling in the pathogenesis of vascular malformations (Figure 1) (75–78). Based on the efficacy of sirolimus, an mTOR inhibitor, in angiomyolipomas associated with tuberous sclerosis complex (79, 80) and Kaposi sarcoma (81), sirolimus was first used in a child with Kaposiform hemangioendothelioma in 2010 (82). Shortly after, a small case series of children with complex vascular malformations and tumors was published, in which all patients demonstrated a significant response and improvement with sirolimus (68). This prompted the first prospective clinical trial that was published in 2016 and confirmed both the efficacy and safety of Sirolimus in the treatment of complex vascular anomalies (83). With the identification of the PI3K/AKT/mTOR pathway as an important driver of somatic overgrowth syndromes (84) and the identification of somatic PI3K mutations within certain vascular malformations (41, 85, 86), PI3K inhibition has become the next major therapeutic target for vascular anomalies (87–89). Future targets will likely be aimed at the RAS/MAPK pathway, where somatic mutations are known to be important in AVMs, capillary malformations, and other aggressive vascular syndromes (10, 44, 55). A list of targeted therapies currently under investigation or in use for vascular anomalies is in Table 11. These discoveries will likely continue to grow at a rapid pace and ultimately inform our classification and management of patients in the clinic. For this reason, it is key for burgeoning vascular anomalies clinical teams to include geneticists as key team members for discovery as well as patient management.

**Table 11:** Current targeted therapies in-use or under investigation for vascular anomalies.

**Drug**	**Mechanism**	**Uses**	**Status**
Sirolimus, everolimus	mTOR inhibition	Complex vascular malformations and vascular tumors	Available, not FDA approved. Multiple ongoing phase I-III trials evaluating use in specific vascular anomalies and also investigating topical use
Trametinib, cobimetinib, selumetinib	MEK inhibition	AVM, possible use in complicated lymphatic anomalies (CCLA, GLA, KLA)	Has been used in some cases without additional therapeutic options (90–92). Upcoming Phase II trial in extracranial AVM
Alpelisib (BYL719)	PIK3CA inhibition	PIK3CA-related overgrowth spectrum disorders (PROS)	Agent is FDA approved for use in breast cancer. Currently under investigation for PROS. Some availability through compassionate use program
ARQ092	AKT inhibition	PROS and PROTEUS syndrome	Under investigation, Phase I/II studies
Propranolol, Atenolol, Timolol (topical)	Beta-blockade	Infantile hemangiomas	Approved as Hemangiol

Trained geneticists and genetic counselors are crucial members of a comprehensive multidisciplinary vascular anomalies team. Detailed discussion of family history, consideration of genetic testing, and review of results is an important part of the comprehensive care of patients with vascular malformations and tumors. Understanding which lesions are likely to be associated with somatic vs. germline changes is key to developing a thoughtful approach to genetic testing. A family history suggestive of an inherited mutation will prompt consideration of germline testing, which can readily be performed by routine phlebotomy. Somatic mutation testing requires sampling the affected tissue, which for most patients requires a skin biopsy with appropriate tissue handling after biopsy is performed. The decision about which genes to screen for and which laboratory to use is made based on a combination of experience, cost, and insurance approval. Current technology available in the field of cancer genomics is likely to be useful as we attempt to further define the genetics of vascular anomalies^.^(93). Members of the genetics and genetic counseling teams are key to providing input on which testing to send as well as interpretation of results. Family should be prepared to receive either definitive positive or negative results, or commonly a variant of uncertain significance with a more nuanced interpretation. Help with interpretation and contextualization of variants of uncertain significance by a geneticist is key as we begin to understand more of the molecular mechanisms and genetic changes in these malformations and tumors. The role of the geneticist and the importance of genetic testing will become increasingly important as the field of vascular anomalies continues to grow, offering new insights into the etiology of these malformations and tumors, and new avenues for improvement in patient care.

## Author Contributions

JD conceived and wrote the manuscript together with AB. DA, FB, and TN contributed critical revision of the article, patient photos for the figure, and final approval of the manuscript. All authors contributed to the article and approved the submitted version.

## Conflict of Interest

The authors declare that the research was conducted in the absence of any commercial or financial relationships that could be construed as a potential conflict of interest.

## References

[1] Mulliken JB Glowacki J. Hemangiomas and vascular malformations in infants and children: a classification based on endothelial characteristics. Plast Reconstr Surg. (1982) 69:412–22. 10.1097/00006534-198203000-00003 7063565

[2] Cox JA Bartlett E Lee EI. Vascular malformations: a review. Semin Plast Surg. (2014) 28:58–63. 10.1055/s-0034-1376263 25045330 PMC4078214

[3] Mulliken JB Burrows PE Fishman SJ. Mulliken and Young's Vascular Anomalies Hemangiomas and Malformations. 2nd ed. New York, NY: University Press (2013). 10.1093/med/9780195145052.001.0001

[4] Enjolras O. Classification and management of the various superficial vascular anomalies: hemangiomas and vascular malformations. J Dermatol. (1997) 24:701–10. 10.1111/j.1346-8138.1997.tb02522.x 9433027

[5] Enjolras O Mulliken JB. Vascular tumors and vascular malformations (new issues). Adv Dermatol. (1997) 13:375–423. 9551150

[6] ISSVA Classification of Vascular Anomalies ©2018. International Society for the Study of Vascular Anomalies (2018). Available online at: “issva.org/classification” (accessed April 23, 2020).

[7] Wassef M Blei F Adams D Alomari A Baselga E Berenstein A. Vascular anomalies classification: recommendations from the international society for the study of vascular anomalies. Pediatrics. (2015) 136:e203–14. 10.1542/peds.2014-3673 26055853

[8] Anusic S Clemens RKJ Meier TO Amann-Vesti BR. Assessment of PTEN-associated vascular malformations in a patient with Bannayan-Riley-Ruvalcaba syndrome. Case Rep. (2016) 2016:bcr2016215188. 10.1136/bcr-2016-215188 27358095 PMC4932365

[9] Limaye N Boon LM Vikkula M. From germline towards somatic mutations in the pathophysiology of vascular anomalies. Hum Mol Genet. (2009) 18:R65–R74. 10.1093/hmg/ddp002 19297403 PMC2657941

[10] Greene AK Goss JA. Vascular anomalies: from a clinicohistologic to a genetic framework. Plast Reconstr Surg. (2018) 141:709e–17e. 10.1097/PRS.0000000000004294 29697621 PMC5922803

[11] Adams DM. Practical genetic and biologic therapeutic considerations in vascular anomalies. Tech Vasc Interv Radiol. (2019) 22:100629. 10.1016/j.tvir.2019.100629 31864536

[12] Queisser A Boon LM Vikkula M. Etiology and genetics of congenital vascular lesions. Otolaryngol Clin North Am. (2018) 51:41–53. 10.1016/j.otc.2017.09.006 29217067

[13] Nguyen H-L Boon LM Vikkula M. Vascular anomalies caused by abnormal signaling within endothelial cells: targets for novel therapies. Semin Intervent Radiol. (2017) 34:233–8. 10.1055/s-0037-1604296 28955112 PMC5615384

[14] Couto RA Greene AK Capillary Malformations. In: Rahbar R Rodriguez-Galindo C Meara JG Smith ER Perez-Atayde AR, editors. Pediatric Head and Neck Tumors: A-Z Guide to Presentation and Multimodality Management. New York, NY: Springer (2014). p. 73–9.

[15] Nguyen H-L Boon LM Vikkula M. Genetics of vascular malformations. Semin Pediatr Surg. (2014) 23:2216. 10.1053/j.sempedsurg.2014.06.014 25241102

[16] Dedania VS Moinuddin O Lagrou LM Sathrasala S Cord Medina FM Del Monte MA. Ocular manifestations of cutis marmorata telangiectatica congenita. Ophthalmol Retina. (2019) 3:791–801. 10.1016/j.oret.2019.03.025 31147303

[17] Shareef S Horowitz D. Cutis Marmorata Telangiectatica Congenita. StatPearls Publishing (2020). 30521220

[18] Thomas AC Zeng Z Rivière JB O'Shaughnessy R Al-Olabi L St-Onge J. Mosaic activating mutations in gna11 and gnaq are associated with phakomatosis pigmentovascularis and extensive dermal melanocytosis. J Invest Dermatol. (2016) 136:770–8. 10.1016/j.jid.2015.11.027 26778290 PMC4803466

[19] Villarreal DJV Leal F. Phacomatosis pigmentovascularis of cesioflammea type. An Bras Dermatol. (2016) 91(5 Suppl. 1):54–6. 10.1590/abd1806-4841.20164516 28300894 PMC5324993

[20] Revencu N Fastre E Ravoet M Helaers R Brouillard P Bisdorff-Bresson A. RASA1 mosaic mutations in patients with capillary malformation-arteriovenous malformation. J Med Genet. (2020) 57:48–52. 10.1136/jmedgenet-2019-106024 31300548

[21] Wooderchak-Donahue WL Johnson P McDonald J Blei F Berenstein A Sorscher M. Expanding the clinical and molecular findings in RASA1 capillary malformation-arteriovenous malformation. Eur J Hum Genet. (2018) 26:1521–36. 10.1038/s41431-018-0196-1 29891884 PMC6138627

[22] Gordon Spratt EA DeFelice T O'Reilly K Robinson M Patel RR Sanchez M. Generalized essential telangiectasia. Dermatol Online J. (2012) 18:13. 10.1172/JCI98589 23286803

[23] Anjaneyan G Kaliyadan F. Angioma Serpiginosum. Treasure Island, FL: StatPearls Publishing (2020). 29083693

[24] Wellman RJ Cho SB Singh P Tune M Pardo CA Comi AM. Gαq and hyper-phosphorylated ERK expression in sturge-weber syndrome leptomeningeal blood vessel endothelial cells. Vasc Med. (2019) 24:72–5. 10.1177/1358863X18786068 30112971 PMC6830577

[25] Sundaram SK Michelhaugh SK Klinger NV Kupsky WJ Sood S Chugani HT. GNAQ mutation in the venous vascular malformation and underlying brain tissue in sturge-weber syndrome. Neuropediatrics. (2017) 48:385–9. 10.1055/s-0037-1603515 28571101 PMC5587372

[26] Boon LM Mulliken JB Enjolras O Vikkula M. Glomuvenous malformation (glomangioma) and venous malformation: distinct clinicopathologic and genetic entities. Arch Dermatol. (2004) 140:971–6. 10.1001/archderm.140.8.971 15313813

[27] Da Costa L O' Donohue MF van Dooijeweert B Albrecht K Unal S. Molecular approaches to diagnose diamond-blackfan anemia: the EuroDBA experience. Eur J Med Genet. (2018) 61:664–73. 10.1016/j.ejmg.2017.10.017 29081386

[28] Vikkula M Boon LM Mulliken JB. Molecular genetics of vascular malformations. Matrix Biol. (2001) 20:327–35. 10.1016/S0945-053X(01)00150-0 11566267

[29] Couto JA Vivero MP Kozakewich HP Taghinia AH Mulliken JB Warman ML. A somatic MAP3K3 mutation is associated with verrucous venous malformation. Am J Hum Genet. (2015) 96:480. 10.1016/j.ajhg.2015.01.007 25728774 PMC4375628

[30] Soblet J Kangas J Nätynki M Mendola A Helaers R Uebelhoer M. Blue Rubber Bleb Nevus (BRBN) syndrome is caused by somatic TEK (TIE2) mutations. J Invest Dermatol. (2017) 137:207–16. 10.1016/j.jid.2016.07.034 27519652

[31] Baigrie D An IC. Blue Rubber Bleb Nevus Syndrome. StatPearls Publishing (2020). 31082129

[32] Tan WH Baris HN Burrows PE Robson CD Alomari AI Mulliken JB. The spectrum of vascular anomalies in patients with PTEN mutations: implications for diagnosis and management. J Med Genet. (2007) 44:594–602. 10.1136/jmg.2007.048934 17526801 PMC2597949

[33] Eng C PTEN hamartoma tumor syndrome. In: Adam MP Ardinger HH Pagon RA Wallace SE Bean LJH Stephens K Amemiya A editors. GeneReviews^®^. University of Washington, Seattle (1993).

[34] Pilarski R. PTEN hamartoma tumor syndrome: a clinical overview. Cancers. (2019) 11:844. 10.3390/cancers11060844 31216739 PMC6627214

[35] Limaye N Wouters V Uebelhoer M Tuominen M Wirkkala R Mulliken JB. Somatic mutations in the angiopoietin-receptor TIE2 can cause both solitary and multiple sporadic venous malformations. Nat Genet. (2009) 41:118–24. 10.1038/ng.272 19079259 PMC2670982

[36] Morrison L Akers A Cerebral cavernous malformation, familial. In: Adam MP Ardinger HH Pagon RA Wallace SE Bean LJH Stephens K Amemiya A editors. GeneReviews^®^. Seattle, WA: University of Washington (1993)

[37] Brouillard P Boon LM Mulliken JB Enjolras O Ghassibé M Warman ML. Mutations in a novel factor, glomulin, are responsible for glomuvenous malformations (“glomangiomas”). Am J Hum Genet. (2002) 70:866–74. 10.1086/339492 11845407 PMC379115

[38] Eerola I Plate KH Spiegel R Boon LM Mulliken JB Vikkula M. KRIT1 is mutated in hyperkeratotic cutaneous capillary-venous malformation associated with cerebral capillary malformation. Hum Mol Genet. (2000) 9:1351–5. 10.1093/hmg/9.9.1351 10814716

[39] Sirvente J Enjolras O Wassef M Tournier-Lasserve E Labauge P. Frequency and phenotypes of cutaneous vascular malformations in a consecutive series of 417 patients with familial cerebral cavernous malformations. J Eur Acad Dermatol Venereol. (2009) 23:1066–72. 10.1111/j.1468-3083.2009.03263.x 19453802

[40] Mellor RH Brice G Stanton AW French J Smith A Jeffery S Mutations in FOXC2 are strongly associated with primary valve failure in veins of the lower limb. Circulation. (2007) 115:1912–20. 10.1161/CIRCULATIONAHA.106.675348 17372167

[41] Limaye N Kangas J Mendola A Godfraind C Schlögel MJ Helaers R. Somatic activating PIK3CA mutations cause venous malformation. Am J Hum Genet. (2015) 97:914–21. 10.1016/j.ajhg.2015.11.011 26637981 PMC4678782

[42] Rodriguez-Laguna L Agra N Ibañez K Oliva-Molina G Gordo G Khurana N. Somatic activating mutations in PIK3CA cause generalized lymphatic anomaly. J Exp Med. (2019) 216:407–18. 10.1084/jem.20181353 30591517 PMC6363432

[43] Lala S Mulliken JB Alomari AI Fishman SJ Kozakewich HP Chaudry G. Gorham-Stout disease and generalized lymphatic anomaly–clinical, radiologic, and histologic differentiation. Skeletal Radiol. (2013) 42:917–24. 10.1007/s00256-012-1565-4 23371338

[44] Barclay SF Inman KW Luks VL McIntyre JB Al-Ibraheemi A Church AJ. A somatic activating NRAS variant associated with kaposiform lymphangiomatosis. Genet Med. (2019) 21:1517–24. 10.1038/s41436-018-0390-0 30542204 PMC6565516

[45] Ozeki M Nozawa A Kawamoto N Fujino A Hirakawa S Fukao T. Potential biomarkers of kaposiform lymphangiomatosis. Pediatr Blood Cancer. (2019) 66:e27878. 10.1002/pbc.27878 31207041

[46] Brouillard P Boon L Vikkula M. Genetics of lymphatic anomalies. J Clin Invest. (2014) 124:898–904. 10.1172/JCI71614 24590274 PMC3938256

[47] Joyce S Gordon K Brice G Ostergaard P Nagaraja R Short J. The lymphatic phenotype in noonan and cardiofaciocutaneous syndrome. Eur J Hum Genet. (2016) 24:690–6. 10.1038/ejhg.2015.175 26242988 PMC4930084

[48] Greenlee R Hoyme H Witte M Crowe P Witte C. Developmental disorders of the lymphatic system. Lymphology. (1993) 26:156–68. 8121193

[49] Mansour S Brice GW Jeffery S Mortimer P Lymphedema-Distichiasis Syndrome. In: Adam MP Ardinger HH Pagon RA editors. GeneReviews®. Seattle, WA: University of Washington (1993) 20301630

[50] Alders M Al-Gazali L Cordeiro I Dallapiccola B Garavelli L Tuysuz B. Hennekam syndrome can be caused by FAT4 mutations and be allelic to van maldergem syndrome. Hum Genet. (2014) 133:1161–7. 10.1007/s00439-014-1456-y 24913602

[51] Jeltsch M Jha SK Tvorogov D Anisimov A Leppänen VM Holopainen T. CCBE1 enhances lymphangiogenesis via a disintegrin and metalloprotease with thrombospondin motifs-3-mediated vascular endothelial growth factor-c activation. Circulation. (2014) 129:1962–71. 10.1161/CIRCULATIONAHA.113.002779 24552833

[52] Scheuerle AE Sweed NT Timmons CF Smith ED Alcaraz WA Shinde DN. An additional case of Hennekam lymphangiectasia-lymphedema syndrome caused by loss-of-function mutation in ADAMTS3. Am J Med Genet A. (2018) 176:2858–61. 10.1002/ajmg.a.40633 30450763

[53] McReynolds LJ Calvo KR Holland SM. Germline GATA2 mutation and bone marrow failure. Hematol Oncol Clin North Am. (2018) 32:713–28. 10.1016/j.hoc.2018.04.004 30047422 PMC6128284

[54] Couto JA Huang AY Konczyk DJ Goss JA Fishman SJ Mulliken JB. Somatic MAP2K1 mutations are associated with extracranial arteriovenous malformation. Am J Hum Genet. (2017) 100:546–54. 10.1016/j.ajhg.2017.01.018 28190454 PMC5339083

[55] Al-Olabi L Polubothu S Dowsett K Andrews KA Stadnik P Joseph AP Mosaic RAS/MAPK variants cause sporadic vascular malformations which respond to targeted therapy. J Clin Invest. (2018) 128:1496–508. 10.1016/j.jid.2018.03.765 29461977 PMC5873857

[56] Goss JA Huang AY Smith E Konczyk DJ Smits PJ Sudduth CL. Somatic mutations in intracranial arteriovenous malformations. PLoS ONE. (2019) 14:e0226852. 10.1371/journal.pone.0226852 31891627 PMC6938308

[57] Starke RM McCarthy D Komotar RJ Connolly ES. Somatic KRAS mutation found in sporadic arteriovenous malformations. Neurosurgery. (2018) 83:E14–5. 10.1093/neuros/nyy163 29917132

[58] Kritharis A Al-Samkari H Kuter DJ. Hereditary hemorrhagic telangiectasia: diagnosis and management from the hematologist's perspective. Haematologica. (2018) 103:1433–43. 10.3324/haematol.2018.193003 29794143 PMC6119150

[59] McDonald J Pyeritz RE Hereditary hemorrhagic telangiectasia. In: Adam MP, Ardinger HH, Pagon RA, Wallace SE, Bean LJH, Stephens K, and Amemiya A. editors. GeneReviews^®^. University of Washington, Seattle (1993). Available online at: http://www.ncbi.nlm.nih.gov/books/NBK1351/ (accessed October 25, 2018). 20301525

[60] McGarry ME Long term oncologic surveillance in maffucci syndrome: a case report. J Oncol Sci. (2017) 3:140–4. 10.1016/j.jons.2017.08.003

[61] Cetinkaya A Xiong JR Vargel I Kösemehmetoglu K Canter HI Gerdan ÖF. Loss-of-function mutations in ELMO2 cause intraosseous vascular malformation by Impeding Rac1 signaling. Am J Hum Genet. (2016) 99:299–317. 10.1016/j.ajhg.2016.06.008 27476657 PMC4974086

[62] Ricci KW. Advances in the medical management of vascular anomalies. Semin Intervent Radiol. (2017) 34:239–49. 10.1055/s-0037-1604297 28955113 PMC5615390

[63] Cioffi A Reichert S Antonescu CR Maki RG. Angiosarcomas and other sarcomas of endothelial origin. Hematol Oncol Clin North Am. (2013) 27:975–88. 10.1016/j.hoc.2013.07.005 24093171

[64] Behjati S Tarpey PS Sheldon H Martincorena I Van Loo P Gundem G. Recurrent PTPRB and PLCG1 mutations in angiosarcoma. Nat Genet. (2014) 46:376–9. 10.1038/ng.2921 24633157 PMC4032873

[65] Sardaro A Bardoscia L Petruzzelli MF Portaluri M. Epithelioid hemangioendothelioma: an overview and update on a rare vascular tumor. Oncol Rev. (2014) 8:259. 10.4081/oncol.2014.259 25992243 PMC4419652

[66] Cheung YH Gayden T Campeau PM LeDuc CA Russo D Nguyen VH A recurrent pdgfrb mutation causes familial infantile myofibromatosis. Am J Hum Genet. (2013) 92:996–1000. 10.1016/j.ajhg.2013.04.026 23731537 PMC3675240

[67] Zhao G Zhu M Qin C Liu X Zhao X. Infantile myofibromatosis: 32 patients and review of the literatures. J Pediatr Hematol Oncol. (2020). 10.1097/MPH.0000000000001603 31764512

[68] Hammill AM Wentzel M Gupta A Nelson S Lucky A Elluru R. Sirolimus for the treatment of complicated vascular anomalies in children. Pediatric Blood Cancer. (2011) 57:1018–24. 10.1002/pbc.23124 21445948

[69] Reinhardt MA Nelson SC Sencer SF Bostrom BC Kurachek SC Nesbit ME. Treatment of Childhood Lymphangiomas With Interferon-α. J Pediatr Hematol Oncol. (1997) 19:232–6. 10.1097/00043426-199705000-00010 9201146

[70] Dickerhoff R Bode VU. Cyclophosphamide in non-resectable cystic hygroma. Lancet. (1990) 335:1474–5. 10.1016/0140-6736(90)91512-9 1972254

[71] Léauté-Labrèze C Dumas de la Roque E Hubiche T Boralevi F Thambo JB Taïeb A. Propranolol for severe hemangiomas of infancy. N Engl J Med. (2008) 358:2649–51. 10.1056/NEJMc0708819 18550886

[72] Hagen R Ghareeb E Jalali O Zinn Z. Infantile hemangiomas: what have we learned from propranolol? Curr Opin Pediatr. (2018) 30:499–504. 10.1097/MOP.0000000000000650 29846253

[73] Zhao F Yang X Xu G Bi J Lv R Huo R. Propranolol suppresses HUVEC viability, migration, VEGF expression, and promotes apoptosis by downregulation of miR-4295. J Cell Biochem. (2019) 120:6614–23. 10.1002/jcb.27957 30368887

[74] Sun B Dong C Lei H Gong Y Li M Zhang Y. Propranolol inhibits proliferation and invasion of hemangioma-derived endothelial cells by suppressing the DLL4/Notch1/Akt pathway. Chem Biol Interact. (2018) 294:28–33. 10.1016/j.cbi.2018.08.018 30130526

[75] Shirazi F Cohen C Fried L Arbiser JL. Mammalian target of rapamycin (mTOR) is activated in cutaneous vascular malformations *in vivo*. Lymphatic Res Biol. (2007) 5:233–6. 10.1089/lrb.2007.1012 18370913

[76] Phung TL Ziv K Dabydeen D Eyiah-Mensah G Riveros M Perruzzi C. Pathological angiogenesis is induced by sustained Akt signaling and inhibited by rapamycin. Cancer Cell. (2006) 10:159–70. 10.1016/j.ccr.2006.07.003 16904613 PMC2531257

[77] Perry B Banyard J McLaughlin ER Watnick R Sohn A Brindley DN. AKT1 overexpression in endothelial cells leads to the development of cutaneous vascular malformations *in vivo*. Arch Dermatol. (2007) 143:504–6. 10.1001/archderm.143.4.504 17438183

[78] Arbiser JL Weiss SW Arbiser ZK Bravo F Govindajaran B Caceres-Rios H. Differential expression of active mitogen-activated protein kinase in cutaneous endothelial neoplasms: implications for biologic behavior and response to therapy. J Am Acad Dermatol. (2001) 44:193–7. 10.1067/mjd.2000.111632 11174372

[79] Bissler JJ McCormack FX Young LR Elwing JM Chuck G Leonard J Sirolimus for angiomyolipoma in tuberous sclerosis complex or lymphangioleiomyomatosis. N Engl J Med. (2008) 358:140–51. 10.1056/NEJMoa063564 18184959 PMC3398441

[80] Herry I Neukirch C Debray MP Mignon F Crestani B. Dramatic effect of sirolimus on renal angiomyolipomas in a patient with tuberous sclerosis complex. Eur J Intern Med. (2007) 18:76–7. 10.1016/j.ejim.2006.07.017 17223050

[81] Stallone G Schena A Infante B Di Paolo S Loverre A Maggio G. Sirolimus for kaposi's sarcoma in renal-transplant recipients. N Engl J Med. (2005) 352:1317–23. 10.1056/NEJMoa042831 15800227

[82] Blatt J Stavas J Moats-Staats B Woosley J Morrell DS. Treatment of childhood kaposiform hemangioendothelioma with sirolimus. Pediatr Blood Cancer. (2010) 55:1396–8. 10.1002/pbc.22766 20730884

[83] Adams DM Trenor CC Hammill AM Vinks AA Patel MN Chaudry G. Efficacy and safety of sirolimus in the treatment of complicated vascular anomalies. Pediatrics. (2016) 137:e20153257. 10.1542/peds.2015-3257 26783326 PMC4732362

[84] Keppler-Noreuil KM Parker VE Darling TN Martinez-Agosto JA. Somatic overgrowth disorders of the PI3K/AKT/mTOR pathway & therapeutic strategies. Am J Med Genet Part C. (2016) 172:402–21. 10.1002/ajmg.c.31531 27860216 PMC5592089

[85] Castel P Carmona FJ Grego-Bessa J Berger MF Viale A Anderson KV. Somatic PIK3CA mutations as a driver of sporadic venous malformations. Sci Transl Med. (2016) 8:332ra42. 10.1126/scitranslmed.aaf1164 27030594 PMC4962922

[86] Kurek KC Luks VL Ayturk UM Alomari AI Fishman SJ Spencer SA. Somatic mosaic activating mutations in PIK3CA Cause CLOVES Syndrome. Am J Hum Genet. (2012) 90:1108–15. 10.1016/j.ajhg.2012.05.006 22658544 PMC3370283

[87] Venot Q Blanc T Rabia SH Berteloot L Ladraa S Duong JP. Targeted therapy in patients with PIK3CA-related overgrowth syndrome. Nature. (2018) 558:540. 10.1038/s41586-018-0217-9 29899452 PMC7610773

[88] di Blasio L Puliafito A Gagliardi PA Comunanza V Somale D Chiaverina G. PI3K/mTOR inhibition promotes the regression of experimental vascular malformations driven by PIK3CA -activating mutations. Cell Death Dis. (2018) 9:1–15. 10.1038/s41419-017-0064-x 29352118 PMC5833448

[89] Castillo S Baselga E Graupera M. PIK3CA mutations in vascular malformations. Curr Opin Hematol. (2019) 26:170–8. 10.1097/MOH.0000000000000496 30855339

[90] Lekwuttikarn R Lim YH Admani S Choate KA Teng JMC. Genotype-guided successful treatment of an arteriovenous malformation in a child. JAMA Dermatol. (2019) 155:256–7. 10.1001/jamadermatol.2018.4653 30566190 PMC7186908

[91] Maynard K LoPresti M Iacobas I Kan P Lam S Antiangiogenic agent as a novel treatment for pediatric intracranial arteriovenous malformations: case report. J Neurosurg Pediatr. (2019) 24:673–9. 10.3171/2019.7.PEDS1976 31585413

[92] Li D March ME Gutierrez-Uzquiza A Kao C Seiler C Pinto E. ARAF recurrent mutation causes central conducting lymphatic anomaly treatable with a MEK inhibitor. Nat Med. (2019) 25:1116–22. 10.1038/s41591-019-0479-2 31263281

[93] Siegel DH Cottrell CE Streicher JL Schilter KF Basel DG Baselga E. Analyzing the genetic spectrum of vascular anomalies with overgrowth via cancer genomics. J Invest Dermatol. (2018) 138:957–67. 10.1016/j.jid.2017.10.033 29174369

